# Role of biomarkers in risk stratification of acute coronary syndrome

**DOI:** 10.4103/0971-5916.73419

**Published:** 2010-11

**Authors:** C.M. Nagesh, Ambuj Roy

**Affiliations:** *Department of Cardiology, Cardio-Thoracic Centre, All India Institute of Medical Sciences, New Delhi, India*

**Keywords:** Acute coronary syndrome, brain natriuretic peptide, cardiac biomarkers, CRP, cystatin C, troponin

## Abstract

Diagnosis of acute coronary syndrome (ACS) encompasses a wide spectrum of myocardial ischaemia varying from assuredly benign to potentially fatal. Cardiac biomarkers have had a major impact on the management of this disease and are now the cornerstone in its diagnosis and prognosis. In this review we discuss both the established and the newer emerging biomarkers in ACS and their role in highlighting not only myocardial necrosis but also different facets of the pathophysiology of ACS. The future of cardiac biomarker testing may be in multimarker testing to better characterize each patient of ACS and thus tailor both short-term and long-term therapy accordingly. This novel concept, however, needs to be tested in clinical trials for its incremental value and cost-effectiveness.

## Introduction

Acute coronary syndrome (ACS) is an umbrella term for a wide spectrum of clinical sign and symptoms suggestive of myocardial ischaemia. The ultimate clinical implication of ACS may therefore vary from assuredly benign to potentially fatal. Thus further risk stratification of this syndrome complex is imperative. It has been seen that 50 per cent of patients hospitalized for suspected ACS ultimately leave the hospital with other diagnoses^1^. Further management of ACS is resource-intensive and thus proper risk stratification is mandatory to avoid needless hospitalizations and interventional procedures. The traditional clinical tools for risk stratification such as history, physical examination, and ECG though undoubtedly important may prove to be inadequate in the majority of cases. This has led to the search for circulating markers that better establish diagnosis and thus aid in appropriate and rapid patient triage. The cardiac necrosis markers creatine phosphokinase and its isoenzymes and especially troponin have come to the forefront in the past decade to better identify high-risk individuals suitable for the most resource-intensive treatment. This is reflected in the various management guidelines of ACS where cardiac enzymes are the cornerstone in decision making. In addition, the success and usefulness of these biomarkers has led to intense research in this field resulting in several newer biomarkers emerging on the horizon of clinical use in ACS.

In this review we discuss the role of these cardiac biomarkers; established and emerging in ACS (Table I) and also the growing evidence of support for the use of multiple biomarkers, each representative of a different facet of the pathophysiology of ACS (Table II).

**Table I:** Biomarkers in acute coronary syndrome

Established biomarkers	Emerging biomarkers
Troponin I	Myeloperoxidase
Troponin T	Metalloproteinase
Brain natriuretic peptide (BNP)	Soluble CD40 ligand
NT-Pro BNP	Ischemia modified albumin
C-reactive protein (CRP)	Pregnancy-associated plasma
	protein-A
	Cystatin C
	Fatty acid binding protein
	Placental growth factor (PlGF)

**Table II:** Various biomarkers underscoring different facets of the pathophysiology and outcomes of ACS

*Inflammation*
C-reactive protein
Myeloperoxidase
Matrix metalloproteinase
Soluble CD40 ligand
*Platelet activation*
Soluble CD40 ligand
*Vulnerable plaque*
Pregnancy-associated plasma protein-A
Myeloperoxidase
Placental growth factor
Matrix metalloproteinase
*Myocardial necrosis*
Creatine phophokinase and isoenzymes
Troponin I and T
Fatty acid binding protein
*Ischaemia*
Ischaemia modified albumin
*Pump failure*
Brain natriuretic peptide
NT-pro brain natriuretic peptide

## Established biomarkers

### Cardiac troponin (cTn)

Cardiac troponin is a well established biomarker for diagnosis and prognosis of ACS^2^–^5^. The data for troponins in ACS is robust even at minimally elevated levels. Measurement of cTnT and cTnI is now the crucial step in new diagnostic criteria for MI^6^. With current high quality analytic methods, cardiac troponin measurements are highly sensitive and specific for myocardial injury^7^. In the appropriate clinical setting (high certainty that the troponin is due to acute coronary syndrome) even minor elevations of troponin identify high risk underlying coronary morphology like patients with plaque rupture, large thrombus burden and distal embolisation^8^. These patients clearly benefit from aggressive anti-platelet, anti-thrombotic and revascularization therapy^9^.

cTn typically increases more than 20 times above the upper limit of the reference range in myocardial infarction as compared to creatine kinase-myocardial band (CK-MB) which usually increases 10 times above the reference range. This provides an improved signal - to - noise ratio, enabling the detection of even minor degree of necrosis with troponin. The cTn begins to elevate 3 h from the onset of chest pain in MI. Because of the continuous release, cTn elevation persists for days (cTnI: 7-10 days, cTnT: 10-14 days). This prolonged course of release with troponin is advantageous for the late diagnosis of MI, however, it limits the diagnosis of early reinfarction.

The cardiac troponin especially cTnT pose diagnostic challenges in patients of chronic renal failure^10^,^11^. Frequent cTnT elevations (30 to 70% of end stage renal disease (ESRD) patients compared with <5% in similar patients of cTnI) are seen in patients of renal failure in the absence of clinical suspicion of ACS^10^,^11^. The putative mechanisms for chronic elevation of troponin in chronic renal disease patients include endothelial dysfunction, acute cardiac stretch, microinfarction and left ventricular hypertrophy^12^. However, it is important to understand that in the setting of acute coronary syndrome these patients should be treated as if renal failure were not present^13^as the short term prognostic value of troponin T for cardiovascular event is similar in patients with and without renal failure.

Data comparing the two cTn suggest that cTnI may be slightly more sensitive. However, this may be due to different release kinetics of the two biomarkers and to different limits of detection of the currently available assays^14^. The other advantage of cTnI may be its greater specificity in patients of ESRD. However, the important advantage of cTnT is that due to international patent restrictions there is only one assay for its measurement, thus cTnT demonstrates a high degree of precision at the low end of measurement range and a relatively uniform cut-off concentration. In contrast, at least 18 different commercial assays for cTnI are available leading to considerable variation in the cut-off concentrations in the definition of a myocardial infarction by cTnI values^15^,^16^. Thus, a clinician should be aware of the cTnI cut-off values specifically associated with the particular assay used by the laboratory.

### Brain natriuretic peptide (BNP)

Brain natriuretic peptide is a neurohormone synthesized in ventricular myocardium and released in response to cardiac stretch. NT-ProBNP is the N-terminal fragment of the prohormone BNP. These natriuretic peptides have prognostic value across the full spectrum of acute coronary syndrome patients.

Patients with elevated BNP or NT-proBNP are at significantly increased risk for subsequently developing heart failure and death both in the short- and long-term. This is seen regardless of their troponin levels and even when there is no clinical evidence of heart failure^17^,^18^. The prognostic value of these peptides is over and above the conventional risk factors like age, Killip class and left ventricular ejection fraction. Studies have shown that BNP predicts high risk features in ACS, such as more severe underlying atherosclerosis, left ventricular dysfunction, left ventricular hypertrophy, and the burden of the ischaemic insult^16^. Thus it may be prudent to conclude that in patients with ACS, the higher the BNP, the more severe the haemodynamic insult due to ischaemia and the worse the prognosis.

### C-reactive protein (CRP)

C-reactive protein is a nonspecific inflammatory marker that is released by the liver in response to the acute phase injury. CRP can be measured by multiple assays in acceptable precisions down to or below 0.3 mg/l and most give comparable results (designated as high-sensitive CRP or hsCRP).

CRP in addition to BNP and troponin does appear to provide some additional value in the prognostication of ACS^20^; however, the incremental value is modest. In terms of the association of CRP and ACS it is important to distinguish cases without (unstable angina) and with necrosis (acute MI). In cases of AMI, CRP release is triggered as an acute phase reactant secondary to necrosis and levels of CRP are much higher and these have been correlated with infarct size. Though infarct size is the major determinant of long term prognosis after AMI; mortality has been shown to be related to CRP levels independent of left ventricular systolic function^21^,^22^. In the absence of infarction, CRP levels correlate to the extent of atherosclerosis and some studies have shown that it predicts coronary events in patients of unstable angina independent of troponin levels^23^,^24^. However, a more recent large prospective study showed only a weak association of CRP levels and future coronary events in patients of ACS and even this disappeared once adjusted for other common clinical variables. This study included about two-thirds of AMI patients and one-third unstable angina patients^25^.

Another interesting implication of CRP in ACS has been in terms of treatment: in a study of ACS patients, those with low CRP levels after statin therapy had better clinical outcomes than those with higher CRP levels, regardless of the resultant level of LDL cholesterol. Thus implying that statin therapy in these high risk patients of ACS should be driven not only by the target lipid levels but also the CRP levels achieved^26^.

These data suggest that CRP levels in ACS may be of prognostic significance but their incremental value over conventional factors and biomarkers may be modest.

## Emerging biomarkers

### Myeloperoxidase (MPO)

Myeloperoxidase is a haemoprotein and lysosomal enzyme released from neutrophilic granules and monocytes^27^. MPO is released into the extracellular fluid and general circulation during inflammatory conditions. This enzyme has been associated with oxidation of lipids contained within LDL, dysfunctional HDL and consumption of nitric oxide thus rendering the normally anti-thrombotic endothelial surface thrombogenic via expression of various pro-thrombotic and anti-fibrinolytic factors^28^. MPO elevation has been associated with adverse ventricular modeling after MI and with progression to heart failure^29^. MPO is responsible for fibrous cap disintegration making it a marker of plaque instability and inflammation. A recent study revealed that elevated MPO levels were marker of cardiac death independent of troponins, CRP in patients of ACS thus highlighting its utility in these patients^30^. However, increased MPO is not likely to be specific to cardiac diseases, as activation of neutrophil and macrophages can occur in any infectious, inflammatory or infiltrative disease process.

### Soluble CD40 ligand

Soluble CD40 ligand (sCD40L) is expressed on platelets and released from them on activation. It has biological activity that can trigger an inflammatory reaction in vascular endothelial cells by the secretion of cytokines and chemokines^31^. Membrane bound CD40L and sCD40L forms interact with the CD40 receptor molecule, which is present not only on B cells but also on monocytes, macrophages, and endothelial and smooth muscle cells in atheroma, leading to release of matrix MMPs and subsequent destabilization of the plaque^32^. Thus upregulation of the CD40L system may play a pathogenic role also in triggering ACS.

Increased sCD40L concentrations have been demonstrated in other inflammatory disorders, *e.g*., autoimmune diseases, multiple sclerosis, and inflammatory bowel disease, as well as in stroke, hypercholesterolaemia, and diabetes^32^,^33^.

In OPUS-TIMI16 trial increased sCD40L was associated with a higher risk for future death and recurrent myocardial infarction independent of other variables including cTnI and CRP. Importantly in combination with cardiac troponin I it significantly improved risk prediction for future death and MI^34^. Similarly in the CAPTURE study of ACS, increased sCD40L concentrations were associated with a higher risk of death and non-fatal MI. Notably elevation of soluble CD40 ligand identified the subgroup of patients likely to benefit from anti-platelet treatment with abciximab^35^. Therapeutic benefits of sCD40L were also seen in MIRACL Study wherein patients with acute coronary syndromes and high sCD40L had a significant reduction in the risk of recurrent cardiovascular events with early statin therapy^36^. However, recent studies have flagged doubts on the influence of pre-analytical and analytical conditions on measurement of sCD40L and thus additional studies are warranted before implementing wider clinical use^37^.

### Ischaemia modified albumin

Ischaemia induces a conformational change in albumin, so that it can no longer bind to transitional metals such as cobalt or copper. Using the albumin cobalt binding (ACB) test, the quantum of ischaemia modified albumin can be estimated and this serves as an index of ischaemia.

Ischaemia-modified albumin (IMA) has been shown to be an independent predictor of short- and long-term adverse outcomes over and above conventional known risk in patients with ACS^38^. Increased IMA values may be found in patients with cancer, infections, end-stage renal disease, liver disease, and brain ischaemia also^39^,^40^.

The commercially available IMA test appears to be relatively sensitive for identifying unstable angina. However, the test’s specificity is relatively poor and the assay is cumbersome to use. With greater refinement it may be a useful test in the emergency department (ED) to rule out ischaemia which is more important at that stage.

### Pregnancy-associated plasma protein-A

Pregnancy-associated plasma protein-A (PAPP-A) is a large, zinc binding proteinase produced by different cell types, including fibroblasts, vascular smooth muscle cells, male and female reproductive tissues and belongs to the insulin-like growth factor family.

It is thought to be released when neovascularization occurs and thus may be a marker of incipient plaque rupture. Its level has been shown to be elevated in unstable plaques and in circulation in patients of ACS^41^. In study of patients with angiographically confirmed acute coronary syndrome, elevated serum PAPP-A was a strong independent predictor of death or recurrent MI, even in patients with normal serum troponin T^42^. Thus preliminary data suggest a possible novel role of PAPP-A in identifying vulnerable plaques, however, additional studies are needed. Moreover, standardized assays for PAPP-A are not available.

### Cystatin C

Cystatin C is a low molecular weight basic protein that is freely filtered and metabolized after tubular reabsorption. There is a U.S. Food and Drug Administration-(FDA) cleared assay that is analytically robust. Some studies have revealed the usefulness of the cystatin C as a prognostic marker in heart failure^43^,^44^and acute coronary syndrome^45^. This protein is less influenced by age, gender, and muscle mass than serum creatinine and thus may be better indicator of cardiovascular risk than serum creatinine especially in elderly.

### Fatty acid binding protein

It is one of the proteins which is rapidly released after myocardial infarction and is considered as alternative to myoglobin. It is an extremely valuable marker of myocardial necrosis in the early hours of ACS and more sensitive than CK-MB, CK-MB mass and cTn^46^.

### Placental growth factor (PlGF)

It is one of the families of platelet-derived proteins that function as potent chemoattractants for monocytes and are involved in the regulation of vascular endothelial growth^47^. It has a high homology with vascular endothelial growth factor. Plasma PlGF measurements have been shown to be an independent biomarker of adverse outcome in patients with suspected ACS^48^. Plasma PlGF appears to extend the predictive and prognostic information gained from traditional biomarkers of necrosis, platelet activation, and systemic inflammation, and has great potential as an independent biomarker for plaque disruption, ischaemia, and thrombosis.

## Multiple biomarker testing: will this be the future norm?

The emergence of different biomarkers in ACS provides insight into the varied pathophysiology of this disease. The future of ACS management would probably shift from single to multimarker testing leading to better characterization of each individual case and thus aid to singularize the stratagem of management of each case in the short- and long-term. In a study to assess the role of multi-marker testing cTnI, CRP and BNP were measured in 450 patients of ACS^20^. It was seen that the mortality was independently related to each biomarker tested and there was a near doubling of mortality rate for each additional biomarker that was positive. Similarly the short term and intermediate cardiac event rates were also strongly related to the number of biomarker positive at admission.

The role of the multiple testing of emerging biomarkers over and above that of the currently established ones needs to be tested in a study and more importantly the impact of a biomarker highlighting a specific pathophysiologic mechanism of ACS in tailoring therapy for an individual patient needs to be established. Although there is still a long way till we reach this destination, it is a noble goal, and the desired direction for the future of cardiac medicine.

## Conclusion

Importance of biomarkers, both in diagnosis and prognosis, of ACS is now well established. Biomarkers like troponin, BNP and CRP are in wide clinical use and substantial evidence of their utility in ACS is present. In addition, several newer biomarkers have recently emerged and may soon be in clinical use as these exemplify different facets of the pathophysiology of ACS and thus may have important therapeutic and prognostic implication over and above that of the established biomarkers. Moreover, these biomarkers would be mutually complementary to each other and thus multi-marker testing would help in better characterizing each case of ACS and may be the future norm.
